# Comparison of gait characteristics between clinical and daily life settings in children with cerebral palsy

**DOI:** 10.1038/s41598-020-59002-6

**Published:** 2020-02-07

**Authors:** Lena Carcreff, Corinna N. Gerber, Anisoara Paraschiv-Ionescu, Geraldo De Coulon, Christopher J. Newman, Kamiar Aminian, Stéphane Armand

**Affiliations:** 1 Laboratory of Kinesiology Willy Taillard, Geneva University Hospitals and University of Geneva, 1205 Geneva, Switzerland; 2 Pediatric Neurology and Neurorehabilitation Unit, Department of Pediatrics, Lausanne University Hospital, 1011 Lausanne, Switzerland; 3 Laboratory of Movement Analysis and Measurement, Ecole Polytechnique Fédérale de Lausanne, 1015 Lausanne, Switzerland; 4 Pediatric orthopedics, Geneva University Hospitals, 1205 Geneva, Switzerland

**Keywords:** Paediatric research, Translational research

## Abstract

Gait assessments in standardized settings, as part of the clinical follow-up of children with cerebral palsy (CP), may not represent gait in daily life. This study aimed at comparing gait characteristics in laboratory and real life settings on the basis of multiple parameters in children with CP and with typical development (TD). Fifteen children with CP and 14 with TD wore 5 inertial sensors (chest, thighs and shanks) during in-laboratory gait assessments and during 3 days of daily life. Sixteen parameters belonging to 8 distinct domains were computed from the angular velocities and/or accelerations. Each parameter measured in the laboratory was compared to the same parameter measured in daily life for walking bouts defined by a travelled distance similar to the laboratory, using Wilcoxon paired tests and Spearman’s correlations. Most gait characteristics differed between both environments in both groups. Numerous high correlations were found between laboratory and daily life gait parameters for the CP group, whereas fewer correlations were found in the TD group. These results demonstrated that children with CP perform better in clinical settings. Such quantitative evidence may enhance clinicians’ understanding of the gap between capacity and performance in children with CP and improve their decision-making.

## Introduction

Cerebral palsy (CP) describes a group of motor disorders resulting from early damage to the developing brain^1^. It is the most frequent motor disability in children, with a prevalence of 1.8 per 1000 live births in Europe^2^. Children with CP have heterogeneous clinical profiles and are classified into five levels of severity with the Gross Motor Function Classification System (I: independent walker; II: independent walker with limitations; III: ambulate with walking aids; IV: ambulate with powered mobility; and V: dependent for all mobility)^3,4^. In CP, gait disorders are among the leading limitations, with a negative impact on participation and self-perception^5^. Current management of gait deviations is largely based on assessments of body structures and body functions of individuals measured in clinical settings^6^. ‘Clinical gait analysis’ (CGA) measures multiple gait parameters in order to identify and understand the main causes of gait deviations^7^. Although CGA has become a widely accepted tool in clinical practice, it is not clear whether in-laboratory assessments reflect the usual walking performance of the patients in daily life. Patients are often considered to perform better when walking under clinical supervision to please caregivers^8^, known as the ‘Hawthorne effect’^9^, and thanks to improved concentration in the absence of external distractors requiring additional attention^10^. Integrating unsupervised assessments of the patients’ daily walking into the clinical process could improve clinicians’ understanding of their real behavior and overall difficulties, beyond the observation of functional limitations in a purely clinical setting^10^.

The link between capacity (what an individual *can* do in a standardized environment) and performance (what an individual *does* do in his usual environment)^11^ remains a largely unsolved question^12^. Various interpretations of capacity can be found in the literature. Capacity can be seen as the best possible level of functioning during short tasks (e.g. assessed by the Gross Motor Function Measure (GMFM)^13^ in CP), as the level of functioning during an endurance task (e.g assessed by the 1- or 6-Minute-Walk Test (1MWT or 6MWT)^14^) or as the spontaneous level of functioning during CGA^15^. For the latter, compound kinematic parameters, such as the Gait Deviation Index (GDI) or Gait Profile Score (GPS)^16^, were mostly reported^17–19^. Performance has mostly been assessed by self- or parent-report questionnaires about daily life mobility^3,20^, physical activity habits and activity limitations^21,22^. Thanks to the increasing availability of wearable motion sensors, objective data about performance is now accessible^23^. Daily number of steps, time spent inactive and time spent in moderate-to-vigorous physical activities (MVPA) are common metrics used to quantify motor performance. Considering this high variety of metrics and definitions, no consensus has been found on the link between capacity and performance in children with CP. Capacity seems to exceed performance^24^, however this relationship is not constant over time and across all GMFCS levels^25,26^. Low to moderate correlations between capacity and performance were found in the majority of studies^17–19,27,28^.

Gait characteristics are measured in the context of a walking activity which can be performed in a standardized environment, e.g. during CGA, then called walking capacity, or in a usual environment, then called walking performance. Gait characteristics are related to the body functions (gait pattern functions as classified in the ICF, WHO^29^), in opposition to gait quantity which is rather associated with the amount and intensity of the ambulatory activity. The previously-mentioned studies essentially demonstrated that gait characteristics (GDI, GPS, walking speed) measured in the laboratory cannot predict gait quantity in daily life^30^. To date, data on gait characteristics in daily life settings is lacking, and could bring additional valuable insights into the motor performance of children with CP.

Gait can be described by multiple features since it involves various physiological systems^31^. Distinct domains can depict gait function such as pace, rhythm, variability, asymmetry, postural control, amplitude, etc.^32–34^. In the context of CGA, spatiotemporal, kinematic, kinetic parameters, among others, are commonly assessed. Motion sensors such as Inertial measurement units (IMU) can quantify several of these gait parameters with a good level of accuracy in pathological populations^35–37^. In children with CP, few sensor configurations have been tested. Foot placement accurately estimates spatiotemporal parameters in children with a low level of disability^38^, while sensors on the lower limbs (shanks and thighs) demonstrated better accuracy for speed estimation in children with higher levels of disability (GMFCS III)^39^. A sensor located on the trunk was also found to appropriately measure parameters of postural control^40,41^ and to accurately estimate cadence^42^. IMUs have the potential to assess gait characteristics in real life settings and to enable direct comparisons with gait measured in the laboratory.

The purpose of this study was to compare gait characteristics between laboratory and real life settings on the basis of multiple features representing different aspects of gait, in children with CP and typical development (TD). The comparisons were based on the evaluation of the difference and the association between parameters in both environments at the group level.

## Method

### Participants

This observational cross-sectional study included a convenience sample of patients diagnosed with CP and followed at the Geneva University Hospitals, aged between 8 and 20 years and with a level of gross motor function (GMFCS) between I and III, meaning that they were able to walk independently with or without mechanical assistance. A group of TD children were also recruited, similar in age and sex. The exclusion criteria for both groups were the standard criteria that preclude adequate participation to the requested tasks, such as significant behavioral issues, severe visual disorders, attention deficit or mental age inferior to 8 years. The protocol was approved by and carried out in accordance with the hospital’s institutional ethical committee (Cantonal Commission for Research Ethics of Geneva - CCER-15-176). Informed consent was obtained from a parent, a legal guardian or the participant him/herself (if older than 18 years).

### Measurement protocol

**Figure 1:**
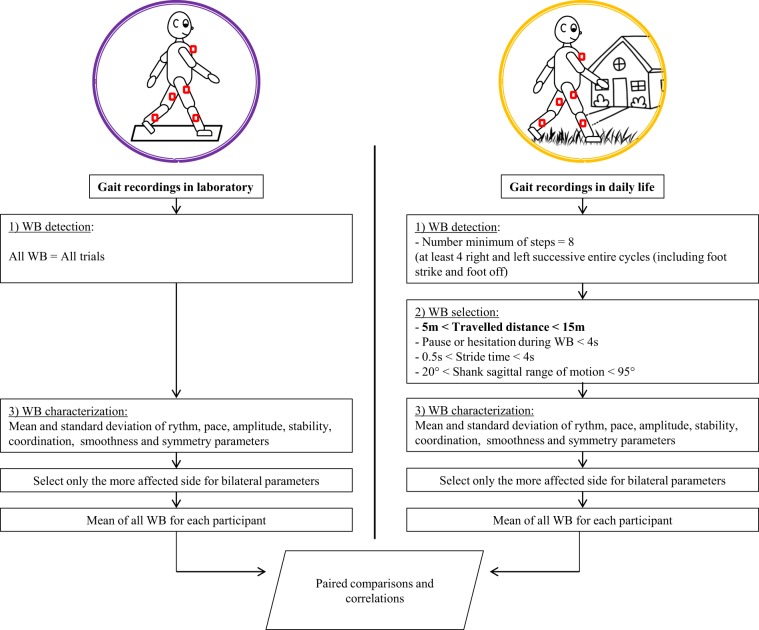
Sensor configuration and flowchart for data processing regarding walking bout (WB) detection, selection and characterization.

This study protocol was twofold. First, the participants performed barefoot standard gait assessments in the laboratory with the instruction to “walk as usual, as if you were in the street”, as in a CGA protocol. Several (between 4 and 10) back and forth walking trials over a 10-meter walkway were performed. Second, the participants were monitored during 3 days including 2 school days and one day of the weekend, for at least 10 consecutive hours. During both assessments, five synchronized IMU-based devices (Physilog4®, GaitUp, Switzerland) were fixed on the lower limbs (shanks and thighs) and on their chest (Fig. 1). Each IMU comprised a triaxial accelerometer (range ± 16 g), and triaxial gyroscope (range ± 1000°/s) with a sampling frequency of 100 Hz. For the in-laboratory measurements, the IMUs were fixed by the investigatorwith hypoallergenic adhesive films (Opsite Flexigrid, Smith & Nephew Medical, UK). At the beginning of each day of daily life measures, the IMUs were placed by the parents or caregivers, who received practical training (as well as a user guide to support them at home) from the investigator for the IMUs management and placement, with hypoallergenic double-sided hydrogel stickies (PAL stickies, PAL Technologies Ltd., UK). The IMUs were also protected from falling with a handmade Elastane sleeve, or under tight pants and socks. At the beginning of the first (laboratory) assessment, a trained investigator measured anthropometric values (shank and thigh lengths) and lower limb muscle strength (using the Medical Research Council testing^43^) for each participant. The delay between the two assessments was of 7 ± 3 months, since the laboratory measurements were performed within an initial technical validation study^39^ and daily life measurements in subsequent reliability^6^ and interventional studies, constrained by school holidays and logistic issues (number of available sensors). None of the children underwent surgery or intensive therapy between both measurements.

### Pre-processing

Laboratory measures: IMU data recorded continuously by all devices was automatically cropped into several walking episodes (corresponding to each back and forth trial on the walkway in the laboratory, i.e. excluding turns). To guaranty reproducible measure and be independent of the IMU location on each segment, lower limbs sensors were automatically aligned with the functional axis of the movement. To this end, assuming that the main angular rotation during gait occurs around the medio-lateral axis of each segment, principal component analysis (PCA) was applied on angular velocity to assess the pitch component of the shanks and thighs rotation^44,45^. For each trial, the norm of acceleration of the chest was computed, to preclude wrong axis selection resulting from potential misalignment of the sensor with regard to the chest.

Daily-life measures: Walking episodes were detected within the continuous daily recording using the pitch angular velocity of both shanks based on the method described by Salarian *et al*.^46^. The 3D signal of the longest detected walking bout (WB) was used to determine the PCA coefficients; then, axis alignment was performed on the entire signal to extract the pitch angular velocity, as for the laboratory assesment. The norm of acceleration of the chest was also computed for each WB. Only WBs with a minimum of 8 steps were considered for the next steps, to preclude from the inclusion of too short WB.

### Walking bout selection

Since in the laboratory the instruction was to walk continuously along a 10-meter walkway, in this study we included daily life WBs with a travelled distance corresponding to approximately 10 m (from 5 to 15 m), without breaks or aberrant gait cycles (resulting from false positive detected gait cycles), in order to represent similar conditions.

Thresholds for break and aberrant gait cycle definitions were set based on data collected during the standard laboratory assessment at various speeds, with the same study participants. Details are provided in the Appendix.

Included WBs were then further characterized as described in the next section, and compared with the WBs in the laboratory. An overview of data processing is presented in Fig. 1.

### Walking bout characterization

This study sought to characterize gait function through several aspects, called ‘domains’. For each domain, a very large number of variables could have been considered so we chose the ones that were the most used in the literature, and we applied some ‘rules’. The common rules for parameters inclusion were: to avoid duplication of parameters from one domain to another, and to avoid redundancy of parameters within the same domain^33^. Therefore, based on the literature^32,33,47^ and the potential of IMU data, eight gait domains with the corresponding parameters were defined, as follows. For bilateral parameters, only the more affected side (based on muscular strength of the lower limbs) of children with CP, and arbitrarily, the left side for TD children, was selected for WB characterization.

#### Rhythm

Following the detection of right and left ‘foot strike’ and ‘foot off’ events on the shank pitch angular velocity signals (using the method described by Salarian *et al*.^46^), the following temporal parameters of gait were computed: stride time and stance time (as a percentage of stride time). Swing time and cadence were redundant information (since swing time = (stride time – stance time) and cadence = (120/stride time)), so they were not reported.

#### Pace

Stride length was computed from the pitch angular velocity of the shanks and thighs, based on the double pendulum model introduced by Aminian *et al*.^46,48^. This model uses thigh and shank lengths and orientations (computed by the numerical integration of pitch angular velocities) at foot strike and foot-off instants of time. Walking speed was computed as the ratio between stride time and stride length.

#### Amplitude

The knee flexion-extension angle was computed by the difference between shank and thigh angles^46^. This parameter has been described as highly representative of the gait pattern of children with CP^49–51^. The ranges of motion (ROM) over the gait cycle were computed.

#### Stability

The time of double support (when both feet are on the ground) as a percentage of the stride time was computed. This outcome was found to be increased in the children with CP in order to ensure better stability^38^. Furthermore, for stability assessment, the standard deviation of the norm of chest acceleration was computed (Trunk Acc._SD_)^52^. Standard deviation (dispersion relative to zero) was chosen instead of root mean square (dispersion relative to the mean) to remove the gravity component^52^.

#### Coordination

The walk ratio was described as a simple index for temporal and spatial coordination description, independent from walking speed^53,54^ and as an outcome measure for treatments aiming at improving motor coordination^55^. Since step length was not computable with our system, the walk ratio as described by Sekiya *et al*.^54^ was computed using the ratio between stride length and cadence. Furthermore, the cyclogram has previously been described as a marker of coordination in subjects with total hip arthroplasty^56^, knee-amputees and adults with CP^57^. The area and the perimeter of the shank-thigh elevation angle cyclogram were computed. The ratio between the cyclogram perimeter and the root mean square of the cyclogram area was used as a coordination parameter^58^.

#### Smoothness

The smoothness of a movement can be affected by spasticity which is a major issue in CP^59^. Higuchi’s fractal^60^ dimension was used for this purpose in children with hemiplegia to assess the smoothness/roughness of the affected upper limb^61^. Fractal dimension was computed on the shank pitch angular velocity time series, for each gait cycle.

#### Variability

Gait variability is known to be higher in children with CP than TD peers in a clinical context^38,62^. Inter-cycle variability was computed as the standard deviation for the rhythm and pace parameters^63^. The standard deviation was preferred to the coefficient of variation (=standard deviation/mean x 100) for better interpretability and to avoid extreme values due to low means^64^.

#### Asymmetry

Symmetry is a good indicator of gait efficiency^32^ and is particularly impaired in the population with unilateral CP^40^. The symmetry index^65^ was computed for the stance time and knee angle since they represent step parameters (in opposition with stride parameters which combine right and left sides). The symmetry index was chosen since it was demonstrated to be the most sensitive to detect gait asymmetry from spatiotemporal parameters in healthy subjects, and the most commonly used in studies reporting symmetry^65^. The limp, representing the difference between the initial and terminal double support, was also computed^46^.

### Data analysis

Non-parametric tests were used in light of the small sample size. Paired Wilcoxon tests were used to compare the medians of laboratory and daily-life gait parameters. Spearman’s correlation coefficients (rho) were computed between the laboratory and the daily-life gait parameters. Altman’s guidelines were used to interpret the correlation: poor, if rho < 0.20; fair, if 0.20 ≤ rho < 0.40; moderate, if 0.40 ≤ rho < 0.60; good, if 0.60 ≤ rho < 0.80; and very good, if rho ≥ 0.80^66^. Alpha was set at 0.05, and the results with Bonferroni’s correction were also presented. Effect size was computed by dividing the Wilcoxon test statistic by the square root of the number of observations, as suggested by Pallant *et al*.^67^.

## Results

### Participants’ characteristics and ambulatory activity

Fifteen children with CP and 14 children with TD were included. One child with CP – GMFCS III had only one WB exceeding 5 m so we chose to exclude her for further analysis. Therefore, the remaining participants’ characteristics are presented in Table 1. The dominant clinical presentation of participants with CP was spastic diplegia (n = 12) and 50% of them needed a walking aid (crutches or walker) to ambulate in the community.

**Table 1:** Participants characteristics and proportion of included daily life WB.

	CP (n = 14)				TD (n = 14)
Age (years)	12.6 [11.4–13.9]				12.3 [11.5–14.5]
Height (m)	1.51 [1.38–1.60]				1.57 [1.47–1.66]
Weight (kg)	43.5 [36.0–50.5]				45.7 [37.7–57.0]
Sex (number of girls)	8				8
	ALL (n = 14)	GMFCS I (n = 6)	GMFCS II (n = 3)	GMFCS III (n = 5)	
Number of detected WB	211 [113–238]	237 [224–271]	183 [139–279.5]	113 [90–113]	335 [265.5–499]
Median distance travelled / WB (m)	6.4 [4.9–7.3]	13 [12–14]	4.9 [4–7]	5.0 [4.1–5.4]	12 [12–14]
Maximal distance / WB (m)	209.4 [48.8–433.1]	420.9 [363.4–464]	322.4 [185.5–505.7]	47.6 [37.7–50.0]	558.7 [375.6–658.3]
Number of included WB (% of detected WB)	30.3 [28.6–35.6]	31.7 [29.7–36.4]	28.5 [20.6–30.6]	30.0 [28.9–36.3]	31.5 [27.4–34.3]

Results are presented as medians [IQR] of the group.

The number of detected WB, the median and maximal distance per WB are shown in Table 1. We observed that children with CP – GMFCS II and III walked less than 5 m in most of their daily WB. The number of WB included following our criteria of selection (i.e. 5 to 15 m) corresponded approximately to 30% of the detected WB for each group.

### Laboratory versus daily life

**Figure 2:**
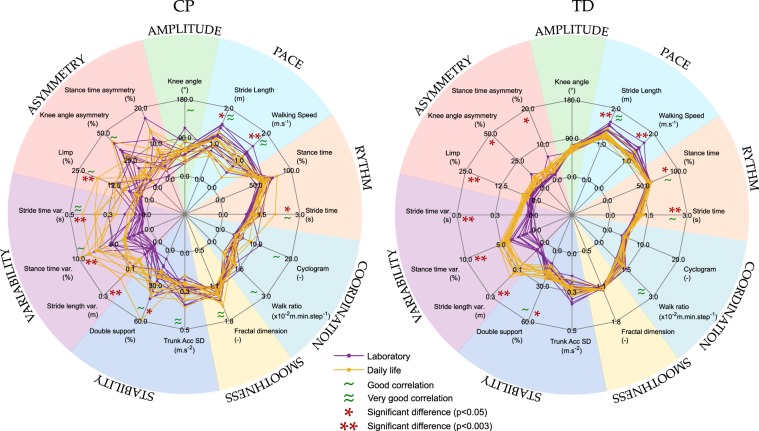
Radar plots presenting the 16 gait parameters (8 gait domains) assessed in laboratory (purple curves) and in daily-life (yellow curves) for the CP and the TD groups. Each curve represents a participant. Significant differences at the group level between in-laboratory and daily-life gait parameters are marked with *if p < 0.05 and with **if p < 0.003 on the corresponding axis. Significant and good correlation (p < 0.05, rho > 0.61) are marked with ~ and very good correlations (p < 0.003, rho > 0.81) are marked with ≈ on the corresponding axis.

The results of the comparisons between gait parameters in laboratory and in daily life are presented in Table 2 and illustrated in Fig. 2 with radar plots for each group. Scatterplots for each parameter, with the distinction of the 2 groups (CP and TD) and the GMFCS levels can be found as Supplementary Fig. S1. There was a high inter-subject heterogeneity within the CP group for both settings as represented on Fig. 2.

**Table 2:** Laboratory and daily-life based gait parameters for each group.

	Variable	Laboratory		Daily life		Paired comparison			Correlation	
		median	IQR [Q1:Q3]	median	IQR [Q1:Q3]	p-value	es	95% CI	rho	p-value
CP (n = 14)	RYTHM									
	Stride time (s)	1.03	[0.98:1.18]	1.25	[1.18:1.32]	**0.004**	0.501	[−0.25:−0.09]	0.72	**0.005**
	Stance time (%)	58.95	[56.83:62.77]	62.52	[58.13:65.21]	0.119	0.223	[−5.51:0.48]	0.53	0.057
	PACE									
	Speed (m.s^−1^)	1.15	[0.84:1.27]	0.91	[0.65:1.01]	0.002*	0.553	[0.09:0.26]	0.85	<0.001*
	Stride length (m)	1.09	[0.93:1.30]	1.12	[0.79:1.14]	**0.011**	0.435	[0.03:0.18]	0.88	<0.001*
	AMPLITUDE									
	Knee angle (°)	53.97	[45.78:64.25]	60.19	[52.28:63.6]	0.296	0.101	[−7.76:3.12]	0.75	0.003*
	ASYMMETRY									
	Stance time asy. (%)	4.34	[2.66:5.62]	6.05	[4.41:7.88]	0.194	0.163	[−4.5:0.53]	−0.05	0.868
	Knee angle asy. (%)	9.89	[7.61:27.5]	12.47	[9.16:14.39]	0.903	0.246	[−4.37:8.2]	0.56	**0.042**
	Limp (%)	4.43	[3.43:6.97]	8.76	[4.26:11.75]	0.002*	0.535	[−4.74:−1.15]	0.65	**0.014**
	VARIABILITY									
	Stride time var. (s)	0.03	[0.02:0.06]	0.21	[0.12:0.33]	<0.001*	0.693	[−0.22:−0.1]	0.90	<0.001*
	Stance time var. (%)	2.90	[1.51:3.78]	6.17	[3.74:7.26]	<0.001*	0.659	[−4.09:−2.16]	0.74	**0.004**
	Stride length var. (m)	0.04	[0.03:0.05]	0.14	[0.12:0.16]	<0.001*	0.693	[−0.12:−0.08]	0.03	0.928
	STABILITY									
	Double support (%)	22.49	[15.62:26.1]	25.94	[20.05:35.59]	**0.035**	0.342	[−9.5:−0.24]	0.64	**0.017**
	Trunk Acc._SD_ (m.s^−2^)	0.24	[0.20:0.26]	0.2317	[0.20:0.27]	0.626	0.061	[−0.02:0.01]	0.91	<0.001*
	SMOOTHNESS									
	Fractal dimension (-)	1.28	[1.21:1.33]	1.28	[1.20:1.30]	0.903	0.246	[−0.02:0.02]	0.93	**<0.01**
	COORDINATION									
	Walk ratio (×10^-2^ m.min.step^−1^)	1.01	[0.88:1.19]	1.1	[0.94:1.17]	0.855	0.2	[−0.09:0.11]	0.74	**0.004**
	Cyclogram (−)	4.58	[4.11:5.30]	4.49	[4.36:5.10]	0.808	0.164	[−0.54:0.53]	0.74	**0.004**
TD (n = 14)	RYTHM									
	Stride time (s)	1.07	[0.98:1.11]	1.12	[1.08:1.21]	0.001*	0.573	[−0.12:−0.05]	0.75	0.003*
	Stance time (%)	58.14	[57.05:60.46]	59.43	[58.64:60.78]	**0.035**	0.342	[−1.87:−0.23]	0.78	0.002*
	PACE									
	Speed (m.s^−1^)	1.28	[1.18:1.38]	1.15	[1.11:1.19]	0.001*	0.573	[0.08:0.24]	0.29	0.318
	Stride length (m)	1.38	[1.25:1.49]	1.26	[1.20:1.30]	0.001*	0.573	[0.06:0.16]	0.87	<0.001*
	AMPLITUDE									
	Knee angle (°)	66.18	[62.63:66.81]	66.97	[63.44:68.71]	0.542	0.02	[−4.34:2.78]	0.28	0.325
	ASYMMETRY									
	Stance time asy. (%)	1.69	[1.46:2.25]	3.65	[2.90:3.92]	**0.011**	0.435	[−1.94:−0.84]	0.42	0.141
	Knee angle asy. (%)	3.33	[2.89:3.56]	7.40	[5.00:8.05]	**0.005**	0.484	[−4.71:−1.54]	0.15	0.605
	Limp (%)	2.03	[1.62:2.57]	3.49	[3.20:4.017]	0.002*	0.535	[−2.07:−0.95]	0.29	0.318
	VARIABILITY									
	Stride time var. (s)	0.02	[0.02:0.03]	0.10	[0.10:0.11]	<0.001*	0.693	[−0.09:−0.08]	0.07	0.820
	Stance time var. (%)	1.83	[0.83:2.56]	4.52	[4.16:4.83]	<0.001*	0.693	[−3.28:−1.96]	0.20	0.483
	Stride length var. (m)	0.03	[0.03:0.04]	0.15	[0.13:0.17]	<0.001*	0.693	[−0.13:−0.10]	0.35	0.215
	STABILITY									
	Double support (%)	16.48	[14.56:20.05]	18.79	[17.04:21.02]	**0.020**	0.387	[−3.45:−0.38]	0.78	0.001*
	Trunk Acc._SD_ (m.s^−2^)	0.23	[0.21:0.26]	0.25	[0.22:0.26]	0.808	0.164	[-0.04:0.03]	0.35	0.221
	SMOOTHNESS									
	Fractal dimension (−)	1.20	[1.18:1.21]	1.20	[1.19:1.21]	0.54	0.02	[−0.02:0.01]	0.49	0.075
	COORDINATION									
	Walk ratio (×10^-2^ m.min.step^-1^)	1.22	[1.10:1.31]	1.13	[1.00:1.29]	0.07	0.282	[0.00:0.11]	0.81	0.001*
	Cyclogram (−)	4.64	[4.54:5.14]	4.88	[4.73:5.00]	0.39	0.052	[-0.29:0.13]	0.58	**0.033**

Var: variability; Asy: Asymmetry; Acc: acceleration; SD: standard deviation; es: effect size, P-values in bold are < 0.05, and a * is indicated if the level of significance after Bonferroni correction (0.003) is reached.

In both groups, all the parameters belonging to the pace and variability domains were significantly (p < 0.011) different between the laboratory and daily life measures. The variability being higher in daily life while the speed was lower. The difference was more pronounced for TD where also rhythm and asymmetry domains were significantly (p < 0.035) different between the two settings. In both groups, no difference was found for the amplitude, smoothness and coordination domains, while only double support was found increased in the stability domain.

In CP, most of the assessed gait parameters across all domains in daily life had good to very good correlations with the parameters in the laboratory (12/16 parameters with rho ≥ 0.60). The highest correlations (rho ≥ 0.80) were found for speed, stride length, stride time variability, trunk acc._SD_ and fractal dimension. In TD however, correlations were less manifest and fewer parameters were correlated (5/16 parameters with rho ≥ 0.60) between the laboratory and the daily life. No significant correlation was found in the amplitude, asymmetry, variability, and smoothness domains.

## Discussion

The objective of this study was to compare multiple gait parameters between two distinct environments: the laboratory where the participant is sought to demonstrate the best of himself^9^ (which can be seen as ‘walking capacity’), and real life where the actual walking habits (‘walking performance’) can be observed. The main findings were that 1) in contrast to TD, most of the gait parameters of children with CP were correlated between both environments, and 2) for both groups substantial differences were found between the settings for most of the parameters, capacity exceeding performance.

While previous studies also emphasized differences between capacity and performance when comparing laboratory and daily life assessments, our findings suggest a certain correlation between gait parameters obtained in laboratory and daily life conditions, which is in contrast to previous findings^17–19,27,28^. The discrepancy can be explained by the dissimilar definitions and metrics used to reflect walking capacity and performance. When gait characteristics evaluated in the laboratory at highest or spontaneous level of functioning was compared to gait quantity in daily life (e.g. total number of daily steps, % spent in MVPA), no correlation was found, involving that CGA cannot be used to estimate daily life quantity of activity^30^. The strength of our study was to compare (i) the exact same metrics of gait and (ii) during similar length of WB in both environments, and high correlations for the majority of parameters in CP were found. This indicates that a child with CP showing higher values in gait parameters measured in the laboratory most probably shows higher values in the same gait parameters in daily life.

As compared to the CP group, children with TD had fewer correlations between gait parameters in the laboratory and daily life. First, this can be due to the values’ heterogeneity in the CP group, favoring correlations, as illustrated in Supplementary Fig. S1 (for example, walking speed ranged between 0.65 and 1.27 m/s in CP, whereas it ranged between 1.10 and 1.38 m/s in TD). Second, this can be the reflection of better capability of children with TD to adapt their gait to the context. This is in agreement with Gosselin *et al*. who stated that individuals with decreased capacity may have difficulties to efficiently respond to unpredictability^10^.

In general, gait function of children with CP and children with TD changed in the same direction, i.e. for instance, higher variability, lower speed, higher asymmetry, and lower stability in daily life. However, there where less gait parameters with significant differences between the laboratory and daily life in children with CP compared to the TD group. This was mostly due to the heterogeneity among children with CP. In fact, greater variations of the parameters were found in the CP group, than in the TD group. As an example, the stance duration increased by 3.6% but was not significant (p = 0.119) at the CP group level, as compared to 1.3% which was significant (p = 0.035) at the TD group level.

This study was the first to compare laboratory versus daily life gait characteristics using identical metrics belonging to various domains in children. Although mostly correlated, not all the gait characteristics in children with CP revealed to be different between both contexts of walking. The results showed that the amplitude, smoothness and coordination domains were similar between both environments for both groups. High correlations were also found in these domains in children with CP, implying that these domains are inherent to their gait pattern independently of the walking context. Van der Krogt *et al*. had similar findings when simulating an external environment with virtual reality (VR)^68^, and comparing kinematic parameters (amplitude domain) of gait during VR and CGA protocols. The variability and asymmetry of gait were higher in daily life in both groups. This was expected since the environment and tasks are more variable in daily life (curved trajectories, inclined or uneven surfaces, obstacles, dual tasking, etc). However, asymmetry of gait in children with CP did not increase as much as for TD children, so they might have stayed on safer and more regular paths. The stability tended to decrease in daily life in both groups but not as significantly as the variability. This was in line with the study of Tamburini *et al*. which showed that the regularity of gait was highly altered by the testing conditions and environments, whereas the stability was not^69^. Finally, pace and rhythm were influenced by the real life context, especially in the TD group with highly significant increases of stride time and stance duration and decreases of speed and stride length. The decrease of speed was due to both decreased stride length and increased stride time (i.e. decreased cadence) in TD, while in CP increase of stride time (i.e. decreased cadence) was the main cause of slower speed. Results in the rhythm domain were also verified in a recent study of Bisi *et al*. assessing children with TD between 6 and 25 years-old in natural and tandem, reflecting challenging walking^70^.

This was the first study to select daily life WB according to the distance travelled. Indeed, the original purpose was to use comparable conditions, using similar metrics. Several previous studies attempted to compare gait parameters in clinical with free-living settings but did not take the WB properties into account. However, regardless of pathology, WB length has a high impact on gait parameters^47,71^. In a study with patients with Parkinson’s Disease, Del Din *et al*. described that gait characteristics in free-living conditions approximated the values of laboratory setting when the duration of the WB corresponded to the time of the laboratory testing protocol^47^, whereas, for other WB lengths, substantial differences and low to moderate correlations for all gait parameters (14 parameters) were found. Selection of WBs may thus be of high importance when comparing laboratory and daily life gait characteristics. Removing curved gait from the WB selection should also be considered in future studies. In this study, only the pitch axis of the gyroscope was aligned with the mediolateral anatomical axis, since functional calibration tasks were difficult to ask to the children, parents or caregivers during the daily-life measurements. The signals in the two other dimensions, which could have been used to determine turning gait, were not used.

Considering the distance criteria for WB inclusions, about 30% of daily life WBs was found to represent the laboratory conditions (standard for all laboratories performing CGA^72^) in each group. Our results also indicated that these 30% of WBs might represent the longest (in distance) WBs for children with CP, especially those with a higher level of disability (GMFCS II and III), whereas they could represent the median WB distance for children with TD and for children with CP with a low level of disability (GMFCS I). This is in agreement with previous studies stating that children with TD are more active on a daily basis^73^.

The findings of this study should be interpreted in light of its limitations. First, a low number of participants were included, lowering the statistical power of the analyses. To help the readers to interpret the significance of the results, effect sizes were reported and the interpretation of p-values can be adjusted according to Bonferroni corrections. Increasing the sample size could have strengthened the conclusions and allowed to divide the CP group into subgroups of severity of the disability (GMFCS levels or laterality of the impairments). In addition, non-parametric tests were performed due to the low sample size. Confounding parameters such as age, sex, height, and weight, that were not adjusted for the model, may have influenced the correlations. Further work investigating this aspect on a bigger cohort should be undertaken.

Laboratory and daily-life assessments were performed months apart. Even if gait is supposed to be stable at this age, this could have introduced bias due to limited changes in morphology which could induce minor changes in the gait pattern.

Next, in this study, only WBs with similar length than laboratory walking were analyzed. This was the chosen solution to make reasonable comparisons of gait characteristics between two contexts of walking. However, clinicians might not only be interested in « short » WBs, especially for children with GMFCS I. Hence, this kind of assessment is intended to be complementary to gait quantity evaluation. Knowing the qualitative parameters that limit gait quantity on a daily basis could indeed be of high interest, especially for the therapists. This could inform them about which gait characteristics should be improved to potentially augment walking quantity.

Regarding IMUs use, the sensor frame alignment was only based on PCA and no conventional functional calibration was performed since the parents or the caregivers mounted the system during the home-based measurements. The PCA axis alignment may have introduced a small bias especially for the children with GMFCS III where transverse and frontal components in the gait pattern are higher than for non-pathological gait. This may have influenced the results of gait parameters in the amplitude, pace, coordination and asymmetry domains for which the angular velocity rate was used for their computation. In addition, the double pendulum model proposed by Aminian *et al*.^48^ relies on precision of leg dimensions (thigh and shank segments lengths) measurements. Although such a measurement with a tape has proven acceptable validity and reliability, potential sources of error can arise when doing the measures on patients with bone deformities and joint contractures^74^.

Finally, through this study, the feasibility of using IMUs to measure objective parameters of gait function in daily life settings have been confirmed. Children showed an overall good acceptability of wearing the sensors since they did not report major issues. However, among all days of measurements, in 27% of the cases, at least one sensor fixation (PAL stickies, PAL Technologies Ltd., UK) was reported deficient. The problem was fixed by the participants with additional medical tape provided by the investigator. The parents and the caregivers did not report any troubles handling the sensors. Among the total days of measurement, 13.1% were interrupted before reaching 10 h of recording (7h50 in the worst case) by the parent or the caregiver, 9.5% of the measurements were interrupted because of battery loss of at least one sensor (6h30 in the worst case), and 3.6% of the measurements had at least one sensor wrongly switched off at the end of the day. In all of these cases, we cut the data at the minimal time between the 5 sensors, resulting in an average of 11 ± 2 h of analyzed recordings per day. Several improvements need to be carried out to maximize the potential of IMUs, e.g. by minimizing the number of sensors and improving the sensor fixation to increase acceptability and performing a complete sensor calibration to compute absolute angles.

This study highlighted the relevance of wearable gait analysis to improve clinical decision making by considering free-living parameters. Clinical decision making is indeed mainly based on 3D motion analysis performed in laboratory settings, when real-life outcomes are the most determinant for children and their families. Tracking gait function in daily-life thanks to IMUs ensures that the effects of clinical decisions ultimately generalize into daily settings. Moreover, IMUs are nowadays close to provide data equivalent to optoelectronic systems, especially kinematics^75^, but need more validations in pathological populations like CP. IMUs have thus the potential to provide a fast, cost-efficient and especially accessible CGA, which are for now restricted to small selection of clinicians due to high costs in material and resources. To conclude, IMUs are now ready to complement 3D gait analysis, and may eventually replace optoelectronic systems once more validation studies will demonstrate their ability to compute kinematics and kinetics. This will open the possibility to perform CGA not only in gait laboratories but also in local medical care settings.

## Conclusion

Gait characteristics assessed in a clinical context appeared highly associated with gait characteristics in a daily life context in children with CP, which was less evident for children with TD. Most gait characteristics differered between both environments (laboratory vs daily life) in both groups. Parameters assessed in the laboratory exceeded the parameters measured in daily life (increased stride time, decreased speed, increased asymmetry, etc.). The present results proved with objective and quantitative evidence that children with CP perform better in clinical settings. Overall, these exploratory findings emphasized the importance of performance considerations in future clinical research to improve clinicians’ understanding of the gap between capacity and performance in children with CP.

## Supplementary information

Supplementary figure S1.

## Appendix A

### Previous collected data to define thresholds

As part of a larger protocol, gait trials at slow and fast self-selected speeds were recorded in addition to spontaneous gait trials in the laboratory. The extremes values within all participants and all groups (CP and TD) were used to define the thresholds.

- Minimal time to consider a break in WB, was determined as the maximal time found between 2 strides (most probably during the slow trials).
- Aberrant gait cycles were defined from aberrant stride times and shanks sagittal ranges of motion (ROM). Bounds for aberrant stride times were determined from the minimal (most probably during fast trials) and maximal (most probably during slow trials) values of stride time. Similarly, bounds for aberrant shank ROM were determined from the minimal (most probably during slow trials) and maximal (most probably during fast trials) values of stride time.

Values are reported in this Table 3

**Table 3:** Thresholds for break and aberrant gait cycles recognition, defined by data recorded in the laboratory.

Minimal stride time	0.6s		Bounds used for aberrant gait cycle time
Maximal stride time	4s	Thresholds used for break definition	
Minimal shank ROM	25°		Bounds used for aberrant shank ROM
Maximal shank ROM	95°		
