# Supplementary material for: Comparison of gait characteristics between clinical and daily life settings in children with cerebral palsy
Source: Sci Rep. 2020 Feb 7;10:2091. doi: 10.1038/s41598-020-59002-6 (PMC7005861; doi:10.1038/s41598-020-59002-6)
Supplement: Supplementary file 1 — Supplementary figure S1. [file 41598_2020_59002_MOESM1_ESM.pdf]

Supplementary information

Manuscript title: Comparison of gait characteristics between clinical and daily life settings in children with cerebral palsy

Authors: Lena Carcreff [1,2,3,]*, Corinna N. Gerber [2], Anisoara Paraschiv-Ionescu [3], Geraldo De Coulon [1,4], , Christopher J. Newman [2], Kamiar Aminian [3,a], and Stéphane Armand [1,a]

Figure caption

S1 – Scatterplots representing the relationships between gait parameters measured in daily life and in laboratory for children with cerebral palsy (CP) and typical development (TD)

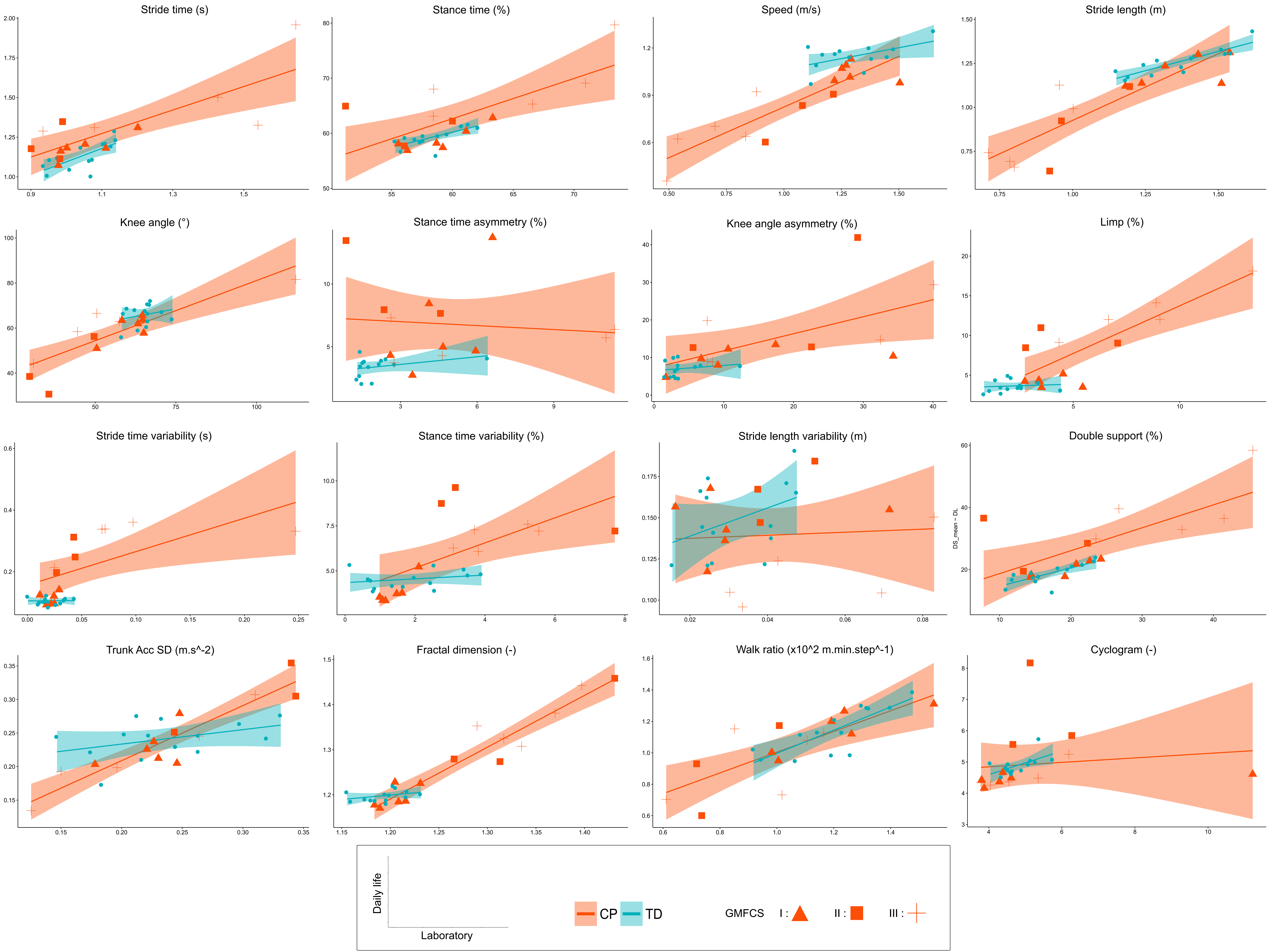
Stride time (s)
Stance time (%)
Speed (m/s)
Stride length (m)
Knee angle (°)
Stance time asymmetry (%)
Knee angle asymmetry (%)
Limp (%)
Stride time variability (s)
Stance time variability (%)
Stride length variability (m)
Double support (%)
DS_mean ~ DL
Trunk Acc SD (m.s^-2)
Fractal dimension (-)
Walk ratio (x10^2 m.min.step^-1)
Cyclogram (-)
Daily life
Laboratory
CP
TD
GMFCS
I :
II :
III :
